# Treatment practices for geriatric type II odontoid fractures – A survey by the European Association of Neurosurgical Societies Spine Section

**DOI:** 10.1016/j.bas.2025.104295

**Published:** 2025-06-14

**Authors:** Ralph T. Schär, Jefferson R. Wilson, Marcel Ivanov, Giuseppe Barbagallo, Yana Petrova, Carla Reizinho, Maria Luisa Gandia González, Enrico Tessitore, Andrzej Maciejczak, Nikolay Gabrovsky, Bart Depreitre, Ehab Shiban, Andreas K. Demetriades, Florian Ringel

**Affiliations:** a Department of Neurosurgery, Inselspital, Bern University Hospital, and University of Bern, Bern, Switzerland; b Department of Surgery, Division of Neurosurgery, St. Michael's Hospital, University of Toronto, Toronto, Canada; c Royal Hallamshire Hospital, Sheffield, UK; d Department of Neurosurgery, University of Catania, Catania, Italy; e Department of Neurosurgery, University Hospital Sofiamed, Sofia, Bulgaria; f Departamento de Neurocirurgia, Hospital Egas Moniz, Unidade Local de Saúde Lisboa Ocidental, Hospital da Luz, Lisbon, Portugal; g Department of Neurosurgery, University Hospital La Paz, Madrid, Spain; h Department of Neurosurgery, Geneva University Hospitals, Geneva, Switzerland; i Department of Neurosurgery, St Lukas Hospital, Tarnów, Poland, Medical Faculty, University of Rzeszów, Poland; j Clinic of Neurosurgery, University Hospital Pirogov, Sofia, Bulgaria; k Department of Neurosurgery, University Hospitals Leuven, Leuven, Belgium; l Department of Neurosurgery, Lausitz University Hospital, Cottbus, Germany; m Department of Neurosurgery, Royal Infirmary Edinburgh, NHS Lothian, Edinburgh, UK; n Department of Neurosurgery, LMU University Hospital, LMU Munich, Germany

**Keywords:** Type II odontoid fracture, European Association of Neurosurgical Societies, Survey, Geriatric population, Spine surgery

## Abstract

**Introduction:**

Controversy exists regarding the optimal management of type II odontoid fractures in the geriatric population. The objective of this study was to determine the current treatment patterns of spine surgeons for geriatric patients (≥70 years) with type II odontoid fractures.

**Research question:**

How much do treatment practices for type II odontoid fractures in the geriatric population differ amongst spine surgeons?

**Methods:**

The European Association of Neurosurgical Societies (EANS) Spine Section distributed a 39-items web-based survey among spine surgeons between July 2024 and February 2025.

**Results:**

A total of 154 responses were collected from 119 neurosurgeons (77.8 %) and 34 orthopedic surgeons (22.2 %). Participants were predominantly from Europe (92.7 %), and 63.2 % have been in practice >10 years. Fracture displacement, comorbidities and age were the most influential factors for decision-making. For non-displaced fractures, 78.8 % of respondents recommended conservative treatment for patients aged 70–80 years, and 83.7 % for those aged 80–90 years. For displaced fractures, 70.9 % preferred surgery for patients aged 70–80 years, whereas this preference decreased to 47.9 % for those aged 80–90 years. Posterior C1-2 fixation was the most common technique for 67.3 % of respondents, and 48.3 % prescribe a collar postoperatively. 51.3 % routinely order CT imaging postoperatively to assess for bony fusion. For conservative treatment, 59.3 % prescribe an external orthosis for 3 months.

**Discussion and conclusion:**

Our survey found both variability and consistency in treatment practices of geriatric type II odontoid fractures, reflecting the ongoing debate and lack of consensus in clinical decision-making.

## Highlights

• Survey shows both variability and consistency in treating geriatric type II odontoid fractures.
• Posterior C1-2 fusion preferred for stability in younger geriatric patients.
• Nonoperative care remains common in frail or high-risk elderly individuals.
• Standardized global guidelines are needed to improve consistency in care.

## 1 Introduction

Odontoid fractures are the most common fractures affecting the cervical spine, and the incidence of these injuries in elderly patients has been increasing due to aging of the population (Grauer et al., 2005; Schoenfeld et al., 2024; Ryan and Henderson, 1992; Golob et al., 2008; Smith et al., 2010). In fact, the oldest-old group of people aged >85 years represents the most rapidly growing segment of the population in developed countries over the past decades (Christensen et al., 2009).

Geriatric patients with type II odontoid fractures pose a dilemma to spine surgeons, and many compromising factors such as frailty, comorbidities and bad bone quality as well as risks of immobilization must be considered when weighing the risk-benefit assessment of surgical vs. conservative treatment. Nonoperative management, while avoiding surgical risks, carries a high risk of nonunion, delayed instability, and complications related to prolonged immobilization, such as overall medical deterioration with increased risk of falls, pneumonia or pressure ulcers from external orthosis. These complications can significantly impact the overall prognosis of elderly patients, who often have limited physiological reserve. Surgical stabilization, on the other hand, offers the advantage of early mobilization and may reduce complications related to prolonged immobilization. However, surgery itself carries inherent risks, particularly in elderly patients. The choice between anterior and posterior surgical approaches is another key consideration.

Despite the multitude of publications on this subject, much controversy remains regarding the optimal management of type II odontoid fractures in elderly patients (Molinari et al., 2012; Denaro et al., 2011; Butler et al., 2010; Chaudhary et al., 2010; Patel et al., 2010; Pal et al., 2011; White et al., 2010). The objective of this study was to determine the differences in current treatment patterns of international spine surgeons for geriatric patients with type II odontoid fractures.

## 2 Materials and methods

### 2.1 Web-based survey and distribution

A multinational cross-sectional survey was conducted among international spine surgeons by the European Association of Neurosurgical Societies (EANS) Spine Section. Recruitment was conducted by distributing the survey link via email or social media with an enclosed letter of invitation and brief explanation of the survey's content and purpose.

The survey consisted of 39 questions, which were formulated in English and subdivided into the following sections: biographical information of the participant (including their training and practice background), decision making for surgical or conservative management, surgical techniques and four sample cases with questions regarding specific management of each case.

For the purpose of this survey, geriatric population was defined as patients of 70 years of age or older.

#### 2.1.1 Statistical analysis

The results of the survey are displayed using descriptive statistics as percentage (%) and standard deviation (SD).

## 3 Results

### 3.1 Demographic data of survey participants

A total of 154 spine surgeons participated in the survey. A detailed overview of the demographic data of the survey's responders is given in Table 1. The data highlights a predominant participation from neurosurgeons (77.8 %), a strong European representation (92.7 %), and a significant proportion of respondents with more than 10 years of practice experience (63.2 %). Most participants work in academic (47.7 %) or partly academic settings (13.7 %), and two thirds of the participants are fellowship-trained in spine surgery (68.6 %).

**Table 1:** Demographic data of 154 survey participants.

Training and working background	N (%)
Specialty	
Neurosurgeon	119 (77.8 %)
Orthopedic surgeon	34 (22.2 %)
Years in practice	
Currently in training	8 (5.3 %)
< 5 years	18 (11.8 %)
5–10 years	30 (19.7 %)
10–20 years	58 (38.2 %)
> 20 years	38 (25.0 %)
Fellowship trained in spine surgery	
Yes	105 (68.6 %)
No	48 (31.4 %)
Region of practice	
Europe	139 (92.7 %)
North America	5 (3.3 %)
South America	3 (2.0 %)
Asia	2 (1.3 %)
Africa	1 (0.7 %)
Working environment	
Academic	73 (47.7 %)
Public	46 (30.1 %)
Private practice	13 (8.5 %)
Both academic and private practice	21 (13.7 %)

#### 3.1.1 Experience and exposure to geriatric odontoid fractures amongst participants

Participants were asked to report the number of geriatric patients (≥70 years) with type II odontoid fractures they manage annually in their practice. The majority of respondents (79.7 %) see fewer than 25 geriatric type II odontoid fracture patients annually, while only a small proportion (6.6 %) manage 50 or more such cases per year (Table 2).

**Table 2:** Estimated exposure to geriatric type 2 odontoid fractures per year of survey participants.

Cases per year	N (%)
<10	64 (41.8 %)
10 to 24	58 (37.9 %)
25 to 49	21 (13.7 %)
50 to 100	7 (4.6 %)
>100	3 (2.0 %)

#### 3.1.2 Factors influencing decision-making for surgical vs. non-surgical management

**Fig. 1:**
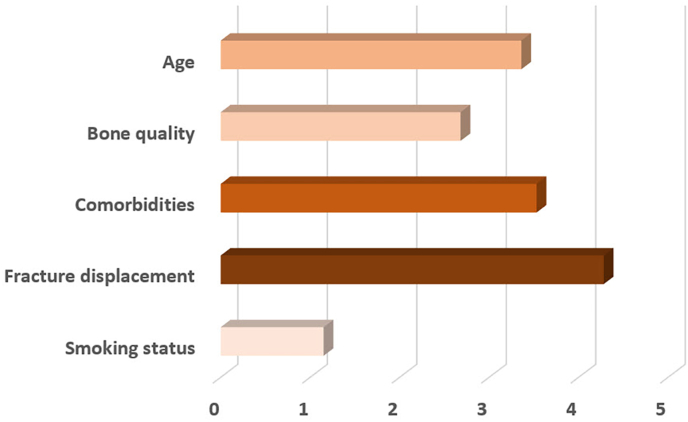
Factors influencing decision-making for surgical vs. non-surgical management.

The survey asked participants to rank five different factors influencing their decision-making process for surgical versus non-surgical management of type II odontoid fractures in geriatric patients. The five factors were age, bone quality, comorbidities, fracture displacement, and smoking status. Responses were scored on a scale from 1 (least important) to 5 (most important). Fracture displacement was the most influential factor, with 56.4 % ranking it as the most important. Comorbidities ranked second in importance, with 36.8 % placing it as the second most critical factor. Patient's age was considered moderately important, with merely 21.4 % ranking it as the top factor. Bone quality was found to be a less significant factor, with 52.1 % ranking it fourth, and smoking status was the least important factor, with 91.5 % of participants ranking it last (Fig. 1).

#### 3.1.3 Typical management strategy for patients aged 70–80 with type II non-displaced (<5 mm) odontoid fractures

In described setting, nonoperative management was recommended by 78.8 % of respondents. Rigid external orthosis was the most common management strategy, chosen by 63.3 %. Soft external orthosis was the less common strategy, with 11.0 % opting for this approach. Open surgical repair with instrumented fusion was selected by 21.2 % of participants. A small minority, accounting for 2.5 %, selected conservative management with no orthosis. Halo vest was the least common strategy, chosen by 0.8 %.

#### 3.1.4 Management strategy for patients aged 80–90 with type II non-displaced (<5 mm) odontoid fractures

Nonoperative treatment was recommended by 87.2 % of participants for this age group. Rigid external orthosis was the most frequently chosen management strategy, selected by 54.7 % of respondents, followed by soft external orthosis (25.4 %). Only 12.8 % of respondents opted for open surgical repair with instrumented fusion. Conservative management with no orthosis was recommended by 6.8 %, and halo vest was not chosen by any of the respondents.

#### 3.1.5 Management strategy for patients aged >90 with type II non-displaced (<5 mm) odontoid fractures

For this age group conservative management with rigid external orthosis (44.9 %), soft external orthosis (31.4 %), or without any orthosis (18.6 %) was clearly favored. Only neurosurgeons saw open surgical repair with instrumented fusion as an option (5.1 %).

#### 3.1.6 Management strategy for patients aged 70–80 with type II displaced (>5 mm) odontoid fractures

For displaced fractures in this age group 70.9 % of respondents suggested open surgical repair and instrumented fusion, followed by rigid external orthosis (23.9 %). Conservative management with no orthosis (2.6 %), soft external orthosis (1.7 %), and halo vest (0.9 %) played minor roles as treatment options.

#### 3.1.7 Management strategy for patients aged 80–90 with type II displaced (>5 mm) odontoid fractures

For this age group 47.9 % chose surgery before rigid external orthosis (42.7 %) as their typical management strategy. Soft external orthosis (7.7 %), and conservative treatment with no orthosis (1.7 %) were the less preferred options. No one chose halo vest as a management strategy.

#### 3.1.8 Management strategy for patients aged >90 with type II displaced (>5 mm) odontoid fractures

Rigid external orthosis was chosen by 56.4 % of all respondents before open surgical repair with instrumented fusion (20.5 %). Soft external orthosis (15.4 %), conservative treatment with no orthosis (6.8 %), and halo vest (0.9 %) were the least often selected management strategies for this age group.

Upfront surgical fixation vs. surgery after failed initial conservative management for geriatric type II odontoid fractures.

Of all participants, a majority of 56.8 % claimed to offer upfront surgery in only up to 25 % of their geriatric type II odontoid fracture patients. In patients with failed initial conservative management, surgical fixation was the typical next treatment step in 0–25 % of cases as responded by 47.9 % of participants. For 31.6 % of respondents surgery would be the typical treatment option after failed initial conservative management in 51–100 % of cases.

Most frequent surgical technique utilized for geriatric type II odontoid fractures, preferred substrate for fusion, and use of postoperative collars or external orthosis.

Participants were asked to rank seven surgical techniques utilized in their practice from most frequent to least frequent. Posterior instrumented fusion with C1 lateral mass screws and C2 pars or C2 pedicle screws was the favored surgical technique for 67.3 % of participants, followed by anterior odontoid screw fixation, posterior fusion with C1-2 transarticular screws, anterior odontoid screw combined with posterior instrumented fusion, posterior wire cerclage, and other not specified techniques.

Preferred substrate for fusion amongst respondents were synthetic bone graft substitute (33.9 %), local autograft (17.8 %), none (14.4 %), allograft (13.6 %), iliac crest autograft (12.7 %), local decortication without graft (6.8 %), or allograft with bone marrow (0.9 %).

Just slightly over half of all participants do not prescribe collars after posterior C1-2 fixation (51.7 %), 18.6 % recommend their patients to wear collars for 3–6 weeks after surgery, 15.3 % for up to 3 weeks, 11.0 % for 6 weeks, and 2.5 % for 12 weeks. Following anterior odontoid screw fixation, 44.1 % of participants do not prescribe collars, 17.8 % do for 3–6 weeks, 16.9 % for up to 3 weeks, 14.4 % for 6 weeks, and 4.2 % for 12 weeks.

How long to wear an external orthosis to manage geriatric type II odontoid fractures, and criteria for terminating external orthosis management.

59.3 % of participants tell their patients to wear an external orthosis for 3 months, 20.3 % recommend 6 weeks, and for 12.7 % of participants duration of wearing an external orthosis is patient-specific depending on symptoms and radiological findings.

A vast majority of 73.7 % of respondents agreed that stopping external orthosis management is both a clinical and radiological decision based on clear improvement of symptoms (e.g. neck pain) and absence of progression of fracture displacement after a predefined period of time (e.g. 6 weeks, 3 months), while evidence of fracture consolidation on CT imaging should not be mandatory. Evidence of fracture consolidation on CT was a key factor for removal of external orthosis for 8.5 % of participants. For 6.8 % of participants termination of external orthosis treatment is a purely clinical decision (clear improvement of symptoms, such as neck pain).

#### 3.1.9 Assessment of fusion after surgery

More than half of all participants (51.3 %) routinely order CT imaging postoperatively to assess for bony fusion. Nearly one quarter of respondents (24.8 %) use both CT imaging and flexion-extension radiographs. 12.8 % claimed not to use any kind of imaging to assess fusion, and 9.4 % only use flexion-extension radiographs.

#### 3.1.10 Pressure ulcers when using external orthosis

For a majority of 57.6 % of all participants pressure ulcers are not a frequent complication encountered when using external orthosis. 22.0 % claimed to frequently see pressure ulcers, but that it does not influence their decision-making regarding surgical vs. conservative management. Another 20.3 % of respondents who frequently encounter pressure ulcers in their patients, favor surgical management in order to avoid this complication.

#### 3.1.11 Sample cases

The survey participants were presented with four sample cases and asked about their typical treatment practices for each case.

**Fig. 2:**
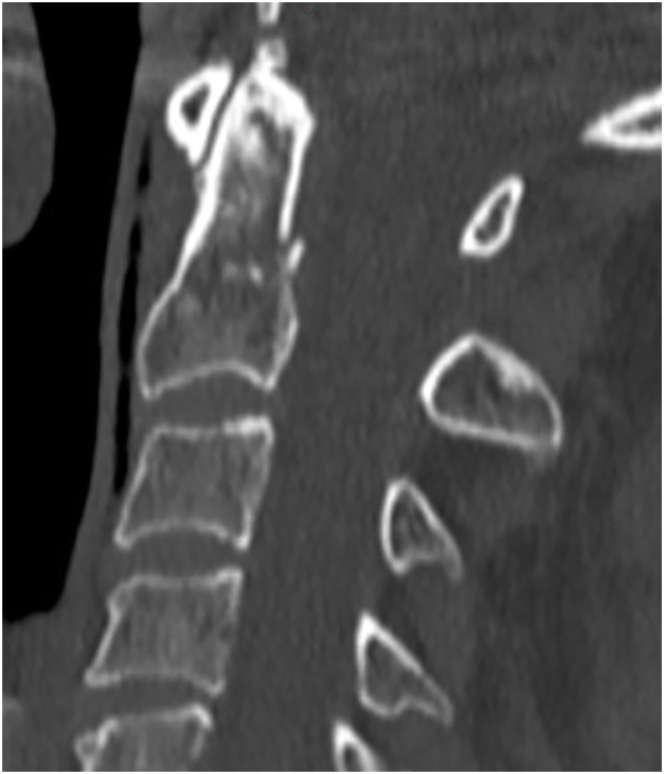
Sample case 1.

Case 1 70 year old women, smoker, arterial hypertension; fall from standing; neck pain, neurologically intact; non-displaced type II odontoid fracture on CT imaging (Fig. 2).

A vast majority of participants (84.5 %) would typically manage this patient non-surgically with a hard collar (59.1 %), with a soft collar (15.5 %), without any external orthosis (7.3 %), or with a halo vest (2.7 %). 14.6 % of respondents would treat this patient surgically with posterior C1-2 fixation (7.3 %) or anterior odontoid screw fixation (7.3 %). Participants who chose non-surgical management were asked about the three main reasons for their decision-making. They were offered the following reasons: belief that surgical fixation will not alter fusion rates, belief that surgical fixation will not improve outcome as compared to non-surgical management, comorbidities, concern surrounding potential for risk of complications, lack of relevant fracture displacement, patient's age, and smoking status. The three main reasons for non-surgical treatment were (1) the lack of relevant fracture displacement, (2) patient's age, and (3) belief that surgical fixation will not improve outcome as compared to non-surgical management. The three main reasons indicated driving the surgical approach were (1) patient's age, (2) presence of fracture displacement, and (3) smoking status.

**Fig. 3:**
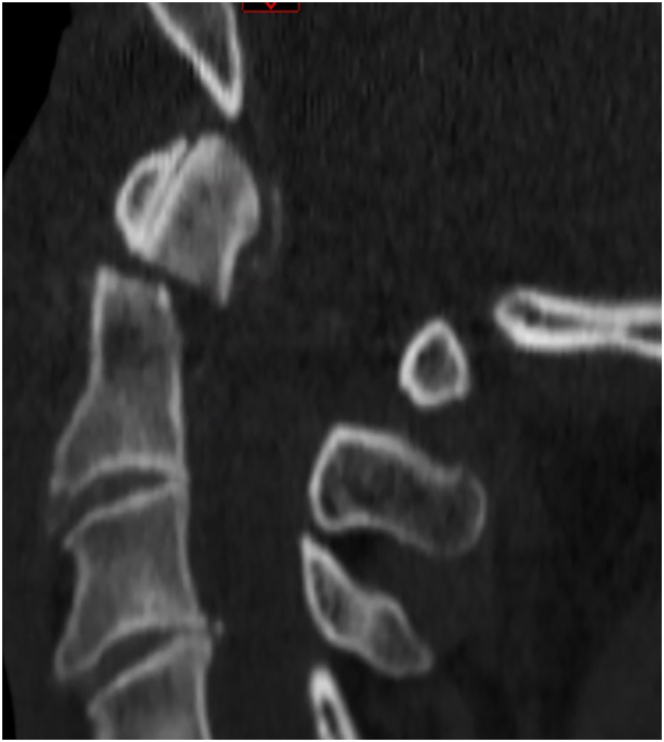
Sample case 2.

Case 2 70 year old man, non-smoker, type II diabetes, arterial hypertension; fell down stairs; neck pain, neurologically intact; 5 mm displaced type II odontoid fracture on CT imaging (Fig. 3).

This case would typically be managed surgically by 79.2 % of the participants (54.5 % posterior C1-2 fixation, 24.8 % anterior odontoid screw fixation). The remainder chose non-surgical management with hard collar (17.8 %), halo vest (2.0 %), or soft collar (1.0 %). The three main reasons for non-surgical management were (1) patient's age, (2) comorbidities, and (3) smoking status. The three main reasons for surgical management were (1) presence of fracture displacement, (2) belief that surgical fixation will improve outcomes as compared to non-surgical management, and (3) patient's age.

**Fig. 4:**
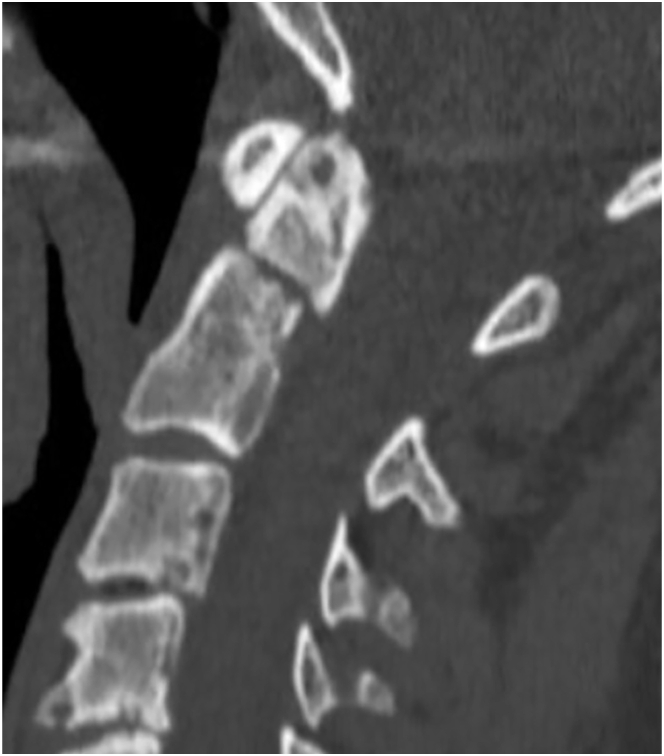
Sample case 3.

Case 3 87 year old man, lives independently, smoker, coronary artery disease, coronary bypass surgery 3 years ago, on dual antiplatelet therapy (ASA, clopidogrel); fall at home; neck pain, neurologically intact; minimally displaced type II odontoid fracture on CT imaging (Fig. 4).

78.5 % of all participants would typically manage this patient non-surgically with a hard collar (53.8 %), soft collar (20.4 %), with no external orthosis (3.2 %), or with a halo vest (1.1 %). The remainder of 20.4 % recommended surgery with anterior odontoid screw fixation (12.9 %) or posterior C1-2 fusion (7.5 %). For one respondent type of management depended on flexion-extension radiograph findings (1.1 %). The three main reasons for non-surgical management were (1) patient's age, (2) comorbidities, and (3) lack of relevant fracture displacement. The three main reasons supporting surgical management were (1) patient's age, (2) presence of fracture displacement, and (3) smoking status.

**Fig. 5:**
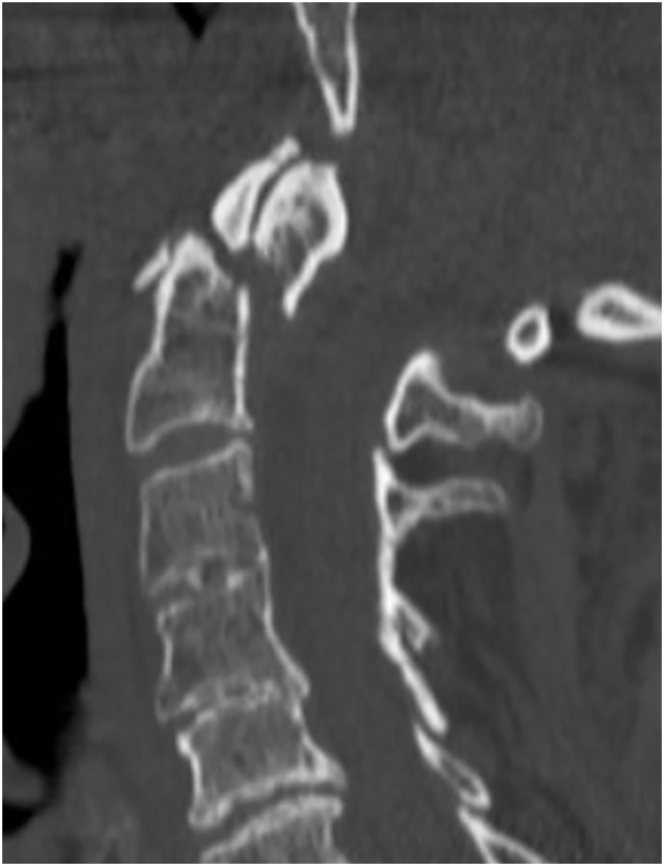
Sample case 4.

Case 4 87 year old woman, non-smoker, nursing home resident, ambulatory with walker, dementia, COPD, type II diabetes; had fall in shower; neck pain, neurologically intact; 7 mm displaced type II odontoid fracture on CT imaging (Fig. 5).

Most participants would typically manage this patient non-surgically (52.2 %) with a hard collar (32.2 %), a soft collar (11.1 %), with no external orthosis (7.8 %), or with a halo vest (1.1 %). Surgical management was preferred in 48.9 % of respondents. Posterior C1-2 fusion (41.1 %) was the favorite approach followed by anterior odontoid screw fixation (6.7 %). The three main reasons for non-surgical management were (1) patient's age, (2) comorbidities, and (3) concern surrounding potential for risk of complications. On the other side (1) presence of fracture displacement, (2) patient's age and (3) belief that surgical fixation will improve outcomes as compared to non-surgical management were the three most common reasons for surgical management.

## 4 Discussion

Our international survey among both orthopedic and neurosurgical spine surgeons provides valuable insights into the current treatment practices for geriatric type II odontoid fractures. Latter has been a hot topic in the last years since international consensus regarding clear treatment patterns for geriatric type II odontoid fractures is largely lacking. Not surprisingly therefore, our survey results confirm that spine surgeons employ a wide range of treatment modalities, including nonoperative management with external immobilization (e.g., soft or hard cervical collars), anterior screw fixation, and posterior cervical fusion. This variability in management is influenced by factors such as patient age, comorbidities, fracture morphology, and surgeon experience. Spine surgeons in our survey were more inclined to offer surgical intervention for displaced fractures and younger geriatric patients (70–80 years) exhibiting displacement, instability, or neurological compromise, with posterior instrumented fusion being the most popular technique. For patients aged 70–80 years with displaced fractures there was an overall agreement amongst participants of nearly 71 % in favor for operative treatment. Conservative approaches seem to be offered more regularly to frail patients >80 years of age and non-displaced fractures, reflecting a tendency toward less invasive strategies in older populations or stable injuries. For non-displaced fractures, there was a growing consensus among survey participants in favor of nonoperative management, with agreement rates increasing from approximately 79 %–95 % across the three age groups: 70–80, 80–90, and over 90 years. The results suggest that rigid external orthosis remains the preferred strategy, but there is an increased inclination toward soft external orthosis and conservative management for older age groups. However, the use of external orthoses, while common in non-surgical management, seems to be less favored postoperatively, especially after posterior C1-2 fixation.

A notable trend observed in our survey is a clear preference for posterior fusion over anterior screw fixation, particularly in older patients with type II odontoid fractures. This shift is likely due to concerns about high rates of nonunion associated with anterior screw fixation, especially in osteoporotic bone. This decision-making is supported by a recent systematic review and meta-analysis on outcomes following anterior versus posterior fixation for odontoid fractures with patients over 60 years of age having significantly higher risk for reoperation and lower odds for fusion (Texakalidis et al., 2023). Additionally, the morbidity associated with prolonged immobilization in cervical collars appears to be a key factor driving the decision toward early surgical intervention in selected cases. Surgeons also reported considerable variation in their use of clinical and radiographic criteria to guide treatment decisions. While some prioritize fracture displacement and patient functional status, others place greater emphasis on bone quality and risk of nonunion. Remarkably, only 51.3 % of participants routinely order CT imaging to assess for bony fusion after surgery, indicating that CT findings do not necessarily correlate with good clinical outcomes. To this point, a recent international prospective comparative study on patients ≥55 years with type II and III odontoid fractures showed similar clinical outcome and fracture healing at 1 year follow-up between surgical and conservative treatment (Huybregts et al., 2024). Interestingly, clinical outcome and fracture union were not clearly associated. The authors emphasized therefore that treatments should prioritize clinical over radiological outcomes. Also, the fear of secondary neurological deficits in conservatively treated patients with odontoid fractures might be unjustified, since the authors recorded no such events.

### 4.1 Clinical and surgical implications

The lack of consensus on optimal treatment for geriatric type II odontoid fractures carries important clinical implications. As these fractures become more prevalent with an aging population, understanding treatment trends and areas of consensus is key to improving outcomes. Management decisions must balance surgical risks against potential complications of conservative care, underscoring the need for a patient-centered approach that considers fracture type, functional status, life expectancy, and individual preferences.

The development of standardized clinical pathways could help guide treatment decisions and improve consistency in care. To address this dilemma, the German Society for Orthopaedics and Trauma published their treatment algorithms in 2023 based on expert consensus and a systematic review in order to provide some guidance for the management of geriatric odontoid fractures (Osterhoff et al., 2023). Nevertheless, the considerable variation in international practice patterns highlighted in our survey underscores the need for multinational guidelines to standardize the management of geriatric odontoid fractures. Current treatment approaches are still largely based on surgeon and regional preferences, as well as institutional protocols rather than consensus-driven recommendations. Standardized guidelines, informed by robust clinical evidence and expert consensus, are essential to ensure that spine surgeons worldwide can provide optimal and consistent care for this vulnerable patient population.

### 4.2 Strengths and limitations

This survey provides an overview on the current state of diverse treatment practices for geriatric type II odontoid fractures in different countries. Our results confirm considerable variations in management and can serve as a reference for further studies and especially as basis for future management guidelines.

The limitations of this work are related to data collection, which requires voluntary participation and subjective evaluation by participants. This may lead to a possible selection bias. Since the survey link was also distributed via social media, an exact response rate cannot be disclosed. In any case, the total of responses is certainly too low to comprehensively represent the international spine surgery community. Also, not all participants responded to every question. This might limit the significance of collective responses to some questions. Lastly, the majority of survey participants were neurosurgeons, with only 22.2 % identifying as orthopedic spine surgeons, and most were based in European countries. This participant distribution may introduce a bias toward neurosurgical and European practices, thereby limiting the generalizability of the findings to the broader global spine community and other clinical settings. Therefore regional differences in treatment practices could not be fully captured. Future studies should aim for a more diverse geographic representation.

## 5 Conclusion

Our international survey of spine surgeons highlights both variability and agreement in treatment practices of geriatric type II odontoid fractures, reflecting the ongoing debate and lack of consensus in clinical decision-making. While nonoperative management was largely favored for non-displaced fractures across all geriatric age groups – and also considered a viable option for displaced fractures in patients over 90 years, particularly in frail individuals – there was a preference for surgical intervention, especially posterior C1–2 fusion, in the younger geriatric population with displaced fractures, to enhance stability and promote early mobilization.The heterogeneity in treatment approaches underscores the need for standardized international guidelines to improve consistency in care and optimize patient outcomes. Future research should focus on comparative studies of surgical and conservative management in different age groups, as well as the development of predictive models to guide treatment decisions based on patient-specific factors such as frailty, bone quality, and comorbidities.

## Declaration of competing interest

The authors declare that they have no known competing financial interests or personal relationships that could have appeared to influence the work reported in this paper.
