# Supplementary material for: Treatment practices for geriatric type II odontoid fractures – A survey by the European Association of Neurosurgical Societies Spine Section
Source: Brain Spine. 2025 Jun 14;5:104295. doi: 10.1016/j.bas.2025.104295 (PMC12210293; doi:10.1016/j.bas.2025.104295)
Supplement: Multimedia component 1 [file mmc1.pdf]

Biographical Information

1. **What is your specialty?**
   Mark one answer only.

   - o Neurosurgeon
   - o Orthopedic surgeon
   - o Trauma surgeon
   - o Other: _________________ *(text box answer)*

2. **How long have you been in practice?**
   Mark one answer only.

   - o I’m still in training to become a spine surgeon
   - o < 5 years
   - o 5-10 years
   - o 10-20 years
   - o > 20 years

3. **Are you fellowship trained in spinal surgery?**
   Mark one answer only.

   - o Yes
   - o No

4. **Which country do you practice in?**
   Mark one answer only.

   - o Drop-down list of countries

5. **Working environment**
   Mark one answer only.

   - o Academic setting
   - o Non-academic public hospital
   - o Private practice
   - o Both

6. **In your practice, how many geriatric (≥ 70 years) type II odontoid fracture patients do you see and manage over the course of a year?**
   Mark one answer only.

   - o 0-10
   - o 10-25
   - o 25-50
   - o 50-100
   - o > 100

Questionnaire

7. Please rank the following factors based on its influence on your decision-making process for surgical vs. non-surgical management of type II odontoid fractures in geriatric patients (≥ 70 years).
Mark one answer only. 1 = most important; 5 = least important

	1	2	3	4	5
Age	O	O	O	O	O
Bone quality	O	O	O	O	O
Comorbidities	O	O	O	O	O
Displacement of fractures	O	O	O	O	O
Smoking status	O	O	O	O	O

8. What is your typical management strategy for a patient between 70-80 years old with type II non-displaced (< 5mm) odontoid fracture?
Mark one answer only.

- o Conservative, no orthosis
- o Rigid external orthosis
- o Soft external orthosis
- o Halo
- o Open surgical repair with instrumented fusion

9. What is your typical management strategy for a patient between 80-90 years old with type II non-displaced (< 5mm) odontoid fracture?
Mark one answer only.

- o Conservative, no orthosis
- o Rigid external orthosis
- o Soft external orthosis
- o Halo
- o Open surgical repair with instrumented fusion

10. What is your typical management strategy for a patient > 90 years old with type II non- displaced (< 5mm) odontoid fracture?
Mark one answer only.

- o Conservative, no orthosis
- o Rigid external orthosis
- o Soft external orthosis
- o Halo
- o Open surgical repair with instrumented fusion

11. What is your typical management strategy for a patient between 70-80 years old with type II displaced (≥ 5mm) odontoid fracture?
Mark one answer only.

- o Conservative, no orthosis
- o Rigid external orthosis
- o Soft external orthosis
- o Halo
- o Open surgical repair with instrumented fusion

12. **What is your typical management strategy for a patient between 80-90 years old with type II displaced (≥ 5mm) odontoid fracture?**
Mark one answer only.

    - o Conservative, no orthosis
    - o Rigid external orthosis
    - o Soft external orthosis
    - o Halo
    - o Open surgical repair with instrumented fusion

13. **What is your typical management strategy for a patient > 90 years old with type II displaced (≥ 5mm) odontoid fracture?**
Mark one answer only.

    - o Conservative, no orthosis
    - o Rigid external orthosis
    - o Soft external orthosis
    - o Halo
    - o Open surgical repair with instrumented fusion

14. **Of geriatric type II odontoid fractures seen, what percentage do you estimate receive surgical management/fixation as primary treatment?**
Mark one answer only.

    - o 0-25%
    - o 25-50%
    - o 50-75%
    - o 75-100%

15. **Of geriatric type II odontoid fractures seen, what percentage do you estimate receive surgical management/fixation after failed initial conservative management?**
Mark one answer only.

    - o 0-25%
    - o 25-50%
    - o 50-75%
    - o 75-100%

16. For geriatric odontoid fractures treated surgically, please rank the 5 most frequent surgical techniques utilized in your practice:
Please rank your 5 top techniques from 1 to 5. Techniques not in the top 5 should be left blank. Check all that apply. 1 = most frequent; 5 = least frequent.

	1	2	3	4	5
Posterior instrumented fusion with C1 lateral mass screws and C2 pars screws	O	O	O	O	O
Posterior instrumented fusion with C1 lateral mass screws and C2 pedicle screws	O	O	O	O	O
Posterior instrumented fusion with C1-2 transarticular screws	O	O	O	O	O
Anterior odontoid screw	O	O	O	O	O
Anterior ondontoid screw AND posterior instrumented fusion	O	O	O	O	O
Posterior wire cerclage techniques (i.e. Galie Fusion or Brooks Fusion)	O	O	O	O	O
Other (please specify below)	O	O	O	O	O

Surgical technique to be specified: ____

17. **For geriatric odontoid fractures treated surgically, what is your preferred substrate for fusion?**
Mark one answer only.

- o Iliac crest autograft
- o Local autograft
- o Allograft
- o Synthetic bone graft substitute
- o Local decortication without graft
- o None
- o Other (please specify): ____

18. When you use external orthosis to manage type II odontoid fractures in geriatric patients (≥ 70 years) conservatively, for how long do you tell your patients to where them?
Mark one answer only.

- o 6 weeks
- o 3 months
- o 6 months
- o Patient-specific, depending on symptoms and radiological findings
- o Other: _________________ *(text box answer)*

19. Conservative treatment: What are your criteria for stopping external orthosis management?
Mark one answer only.

- o Clinical decision: Clear improvement of symptoms (e.g. neck pain) after predefined period of time (e.g. 6 weeks, 3 months, 6 months).
- o Radiological decision (I.): Absence of progression of fracture displacement after predefined period of time (e.g. 6 weeks, 3 months, 6 months), evidence of fracture consolidation on CT not mandatory.
- o Radiological decision (II.): Evidence of fracture consolidation on CT after predefined period of time (e.g. 6 weeks, 3 months, 6 months).
- o Both clinical and radiological decision: Clear improvement of symptoms (e.g. neck pain) and absence of progression of fracture displacement after predefined period of time (e.g. 6 weeks, 3 months, 6 months); evidence of fracture consolidation on CT not mandatory.
- o Other: _________________ *(text box answer)*

20. Do you prescribe external orthosis (collars) after posterior C1-2 fixation?
Mark one answer only.

- No
- Yes, for < 3 weeks
- Yes, for 3-6 weeks
- Yes, for 6 weeks
- Yes, for 12 weeks
- Other: _________________ *(text box answer)*

21. Do you prescribe external orthosis (collars) after anterior odontoid screw fixation?
Mark one answer only.

- No
- Yes, for < 3 weeks
- Yes, for 3-6 weeks
- Yes, for 6 weeks
- Yes, for 12 weeks
- Other: _________________ *(text box answer)*

22. Do you routinely order CT imaging or flexion-extension radiographs postoperatively to assess fusion?
Mark one answer only.

- No, I do not use imaging to assess fusion.
- Yes, I use CT imaging.
- Yes, I use flexion-extension radiographs.
- Yes, I use both CT imaging and flexion-extension radiographs.
- Other: _______________ (*Text box answer*)

23. In your practice, are pressure ulcers a frequent complication when using external orthosis?
Mark one answer only.

- No
- Yes, but it does not influence my decision-making regarding surgical vs. conservative management.
- Yes, and I favor surgical management to avoid pressure ulcers in my patients.

Sample Cases

Case #1

- 70 y/o women
- Smoker, HTN
- Fall from standing
- Neck pain
- Neurologically intact
- Non-displaced type II odontoid fracture (CT shown below)

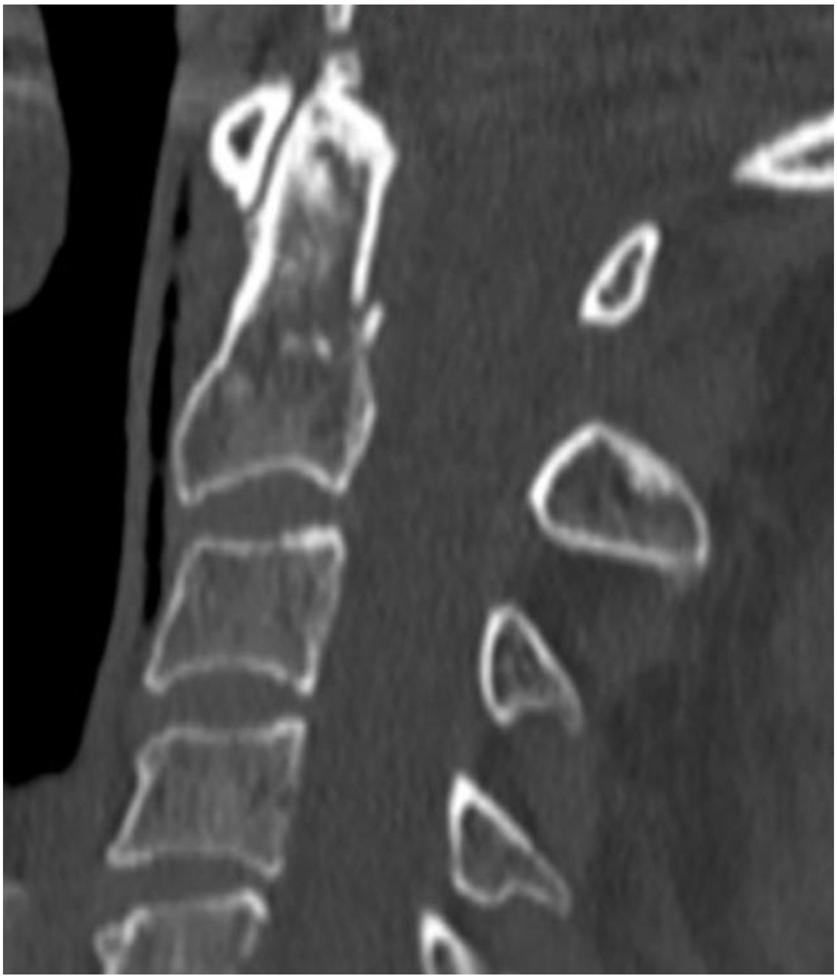

24. How would you typically manage this patient?
Mark one answer only.

a. Non-Surgically with no external orthosis *(Skip to question 26)*
b. Non-Surgically with soft collar *(Skip to question 26)*
c. Non-Surgically with hard collar *(Skip to question 26)*
d. Non-Surgically with halo *(Skip to question 26)*
e. Surgically with posterior instrumented C1-2 fusion *(Skip to question 27)*
f. Surgically with anterior approach (odontoid screw fixation) *(Skip to question 27)*
g. Surgically with another approach not listed above (*Skip to question 25 to specify*)

25. Surgically with another approach not listed above; please specify:

Skip to question 27.

Non-surgical Management

26. Please rank the 3 main reasons for non-surgical management:
Reasons not in the top 3 should be left blank. Check all that apply.
1 = most important; 3 = least important

	**1**	**2**	**3**
Patient's age	O	O	O
Smoking status	O	O	O
Comorbidities	O	O	O
Lack of relevant fracture displacement	O	O	O
Concern surrounding potential for risk of complications	O	O	O
Belief that surgical fixation will not improve outcomes as compared to non-Surgical management	O	O	O
Belief that surgical fixation will not alter rates of fusion	O	O	O

Skip to question 22.

Surgical Management

27. Please rank the 3 main reasons for Surgical management:
Reasons not in the top 3 should be left blank. Check all that apply.
1 = most important; 3 = least important

	**1**	**2**	**3**
Patient's age	O	O	O
Smoking status	O	O	O
Comorbidities	O	O	O
Presence of fracture displacement	O	O	O
Belief that the risk of serious complications is minimal	O	O	O
Belief that Surgical fixation will improve outcomes as compared to non-Surgical management	O	O	O
Belief that Surgical fixation will improve rates of fusion	O	O	O

Case #2

- 70 y/o man
- Non-smoker, type II diabetes, HTN
- Fall down stairs
- Neck pain
- Neurologically intact
- 5 mm displaced type II odontoid fracture (CT shown below)

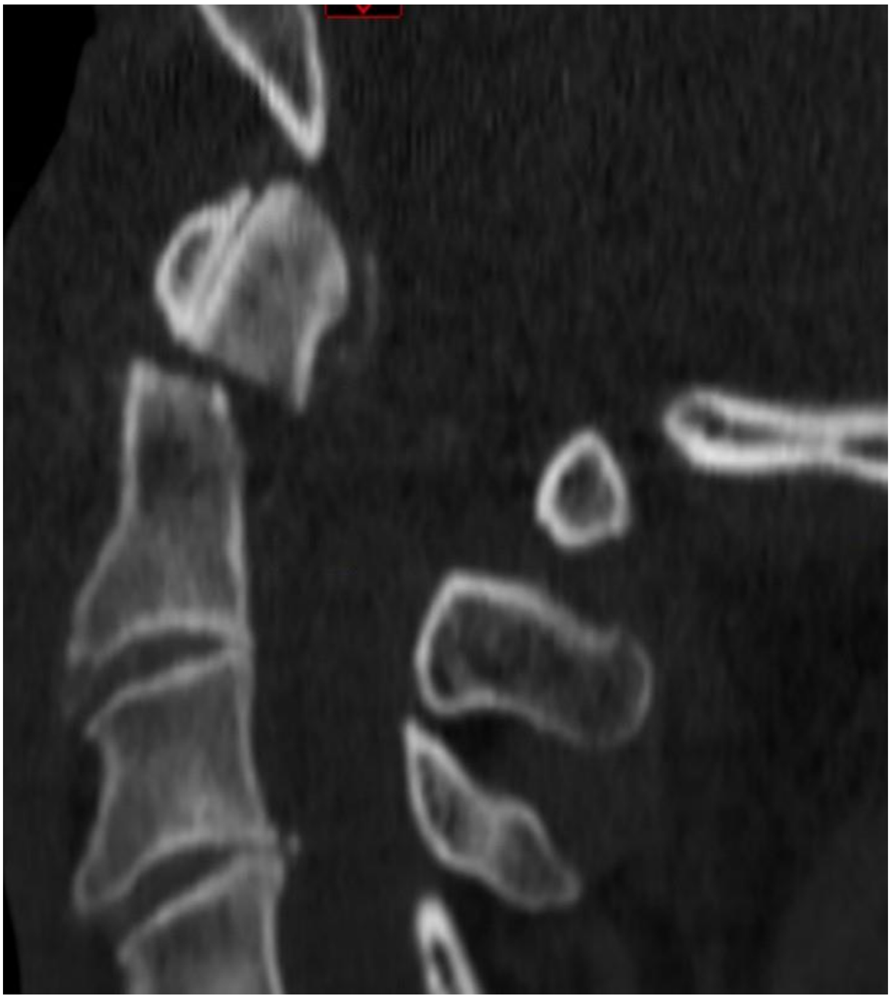

28. How would you typically manage this patient?
Mark one answer only.

- o Non-Surgically with no external orthosis *(Skip to question 30)*
- o Non-Surgically with soft collar *(Skip to question 30)*
- o Non-Surgically with hard collar *(Skip to question 30)*
- o Non-Surgically with halo *(Skip to question 30)*
- o Surgically with posterior instrumented C1-2 fusion *(Skip to question 31)*
- o Surgically with anterior approach (odontoid screw fixation) *(Skip to question 31)*
- o Surgically with another approach not listed above (*Skip to question 29 to specify*)

29. Surgically with another approach not listed above; please specify:

Skip to question 31.

Non-surgical Management

30. Please rank the 3 main reasons for non-surgical management:
Reasons not in the top 3 should be left blank. Check all that apply.
1 = most important; 3 = least important

	**1**	**2**	**3**
Patient's age	O	O	O
Smoking status	O	O	O
Comorbidities	O	O	O
Lack of relevant fracture displacement	O	O	O
Concern surrounding potential for risk of complications	O	O	O
Belief that Surgical fixation will not improve outcomes as compared to non-Surgical management	O	O	O
Belief that Surgical fixation will not alter rates of fusion	O	O	O

Skip to question 26.

Surgical Management

31. Please rank the 3 main reasons for surgical management:
Reasons not in the top 3 should be left blank. Check all that apply.
1 = most important; 3 = least important

	**1**	**2**	**3**
Patient's age	O	O	O
Smoking status	O	O	O
Comorbidities	O	O	O
Presence of fracture displacement	O	O	O
Belief that the risk of serious complications is minimal	O	O	O
Belief that Surgical fixation will improve outcomes as compared to non-Surgical management	O	O	O
Belief that Surgical fixation will improve rates of fusion	O	O	O

Case #3

- 87 y/o man, lives independently, smoker
- Coronary artery disease, bypass surgery (CABG) 3 years ago
- On dual antiplatelet therapy (ASA, clopidogrel)
- Fall at home
- Neck pain
- Neurologically intact
- Minimally displaced type II odontoid fracture (CT shown below)

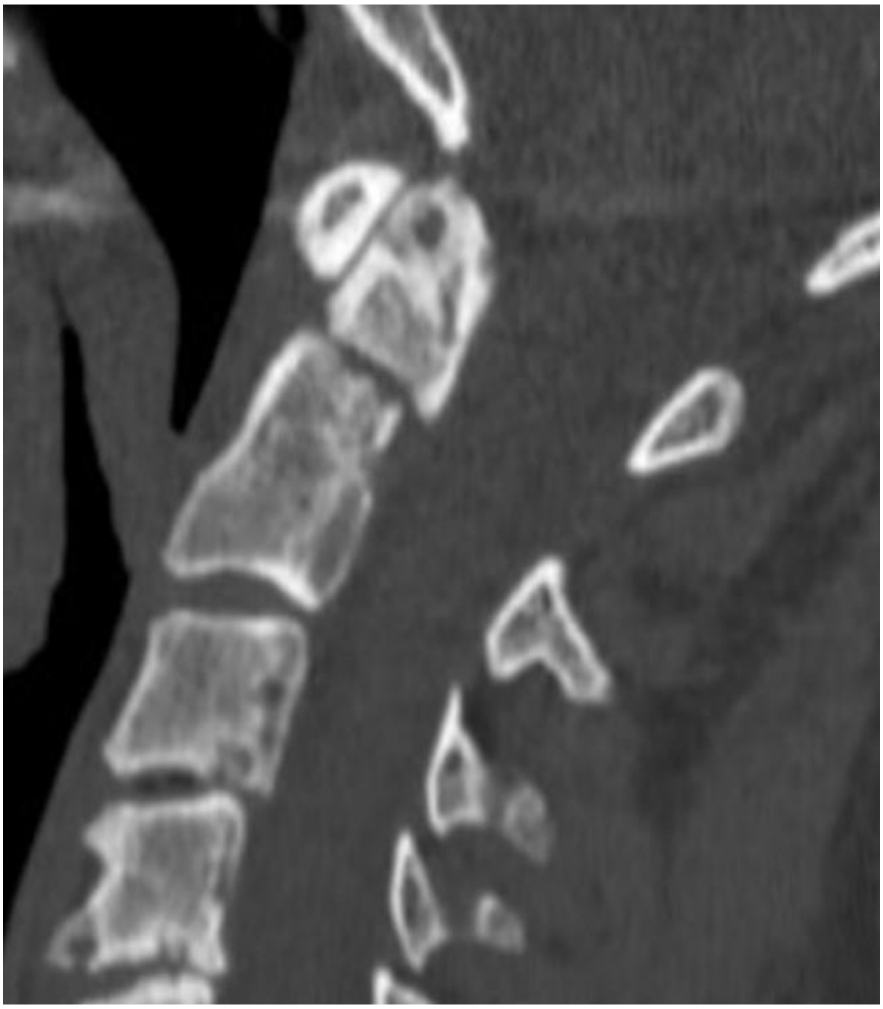

32. How would you typically manage this patient?
Mark one answer only.

- o Non-Surgically with no external orthosis *(Skip to question 34)*
- o Non-Surgically with soft collar *(Skip to question 34)*
- o Non-Surgically with hard collar *(Skip to question 34)*
- o Non-Surgically with halo *(Skip to question 34)*
- o Surgically with posterior instrumented C1-2 fusion *(Skip to question 35)*
- o Surgically with anterior approach (odontoid screw fixation) *(Skip to question 35)*
- o Surgically with another approach not listed above (*Skip to question 33 to specify*)

33. Surgically with another approach not listed above; please specify:

____

Skip to question 35.

Non-surgical Management

34. Please rank the 3 main reasons for non-surgical management:
Reasons not in the top 3 should be left blank. Check all that apply.
1 = most important; 3 = least important

	1	2	3
Patient's age	O	O	O
Smoking status	O	O	O
Comorbidities	O	O	O
Lack of relevant fracture displacement	O	O	O
Concern surrounding potential for risk of complications	O	O	O
Belief that Surgical fixation will not improve outcomes as compared to non-Surgical management	O	O	O
Belief that Surgical fixation will not alter rates of fusion	O	O	O

Skip to question 30.

Surgical Management

35. Please rank the 3 main reasons for Surgical management:
Reasons not in the top 3 should be left blank. Check all that apply.
1 = most important; 3 = least important

	1	2	3
Patient's age	O	O	O
Smoking status	O	O	O
Comorbidities	O	O	O
Presence of fracture displacement	O	O	O
Belief that the risk of serious complications is minimal	O	O	O
Belief that Surgical fixation will improve outcomes as compared to non-Surgical management	O	O	O
Belief that Surgical fixation will improve rates of fusion	O	O	O

Case #4

- 87 y/o woman, non-smoker
- Nursing home resident, ambulatory with walker
- Dementia, COPD, type II diabetes
- Fall in shower
- Neck pain
- Neurologically intact
- 7 mm displaced type II odontoid fracture (CT shown below)

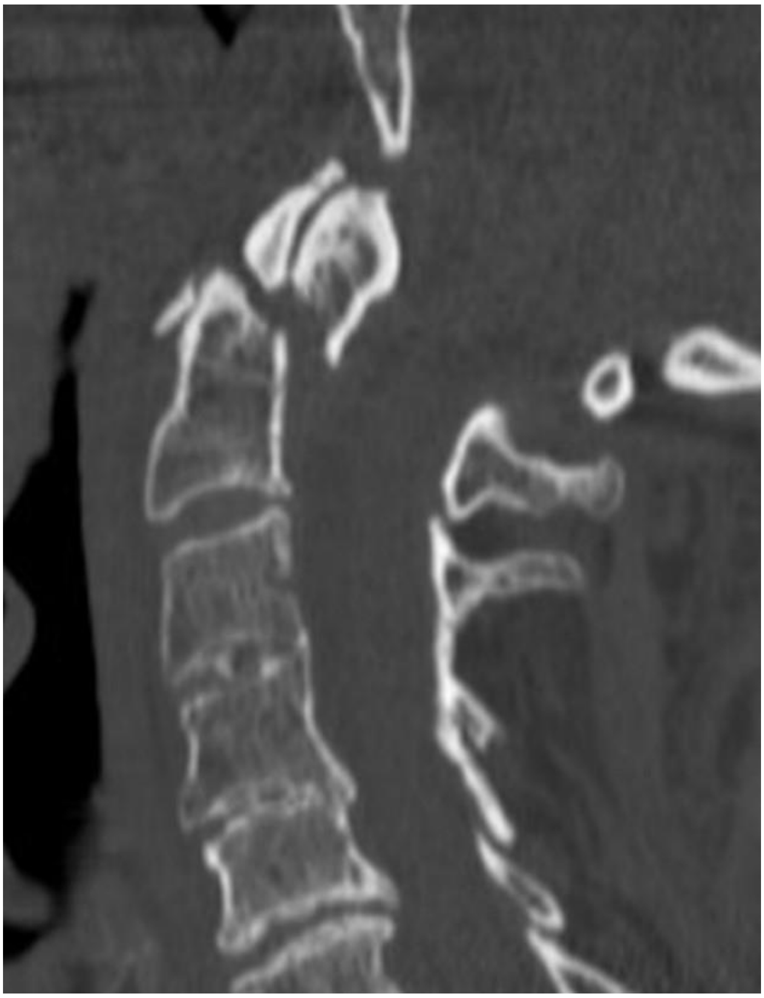

36. How would you typically manage this patient?
Mark one answer only.

- o Non-surgically with no external orthosis *(Skip to question 38)*
- o Non-surgically with soft collar *(Skip to question 38)*
- o Non-surgically with hard collar *(Skip to question 38)*
- o Non-surgically with halo *(Skip to question 38)*
- o Surgically with posterior instrumented C1-2 fusion *(Skip to question 39)*
- o Surgically with anterior approach (odontoid screw fixation) *(Skip to question 39)*
- o Surgically with another approach not listed above (*Skip to question 37 to specify*)

37. Surgically with another approach not listed above; please specify:

Skip to question 39.

Non-surgical Management

38. Please rank the 3 main reasons for non-surgical management:
Reasons not in the top 3 should be left blank. Check all that apply.
1 = most important; 3 = least important

	**1**	**2**	**3**
Patient’s age	O	O	O
Smoking status	O	O	O
Comorbidities	O	O	O
Lack of relevant fracture displacement	O	O	O
Concern surrounding potential for risk of complications	O	O	O
Belief that Surgical fixation will not improve outcomes as compared to non-Surgical management	O	O	O
Belief that Surgical fixation will not alter rates of fusion	O	O	O

You have completed the survey!

Surgical Management

39. Please rank the 3 main reasons for surgical management:
Reasons not in the top 3 should be left blank. Check all that apply.
1 = most important; 3 = least important

	**1**	**2**	**3**
Patient’s age	O	O	O
Smoking status	O	O	O
Comorbidities	O	O	O
Presence of fracture displacement	O	O	O
Belief that the risk of serious complications is minimal	O	O	O
Belief that Surgical fixation will improve outcomes as compared to non-Surgical management	O	O	O
Belief that Surgical fixation will improve rates of fusion	O	O	O

You have completed the survey!
